# *Drosophila Netrin-B* controls mushroom body axon extension and regulates courtship-associated learning and memory of a *Drosophila* fragile X syndrome model

**DOI:** 10.1186/s13041-019-0472-1

**Published:** 2019-05-28

**Authors:** Huaixing Kang, Juan Zhao, Xuan Jiang, Guangxu Li, Wen Huang, Huili Cheng, Ranhui Duan

**Affiliations:** 1 Center for Medical Genetics, School of Life Sciences, Central South University, Changsha, 410078 Hunan China; 2 Changchun Children’ Hospital, Changchun, 130000 Jilin China; 3 Hunan Key Laboratory of Medical Genetics, Central South University, Changsha, 410078 Hunan China; 4 Hunan Key Laboratory of Animal Models for Human Diseases, Central South University, Changsha, 410078 Hunan China

**Keywords:** Mushroom body (MB), *Netrin-B (NetB)*, Axon extension, Fragile X syndrome (FXS)

## Abstract

Mushroom body (MB) is a prominent structure essential for olfactory learning and memory in the Drosophila brain. The development of the MB involves the appropriate guidance of axon lobes and sister axon branches. Appropriate guidance that accurately shapes MB development requires the integration of various guidance cues provided by a series of cell types, which guide axons to reach their final positions within the MB neuropils. Netrins are axonal guidance molecules that are conserved regulators of embryonic nerve cord patterning. However, whether they contribute to MB morphogenesis has not yet been evaluated. Here, we find that *Netrin-B (NetB)* is highly expressed in the MB lobes, regulating lobe length through genetic interactions with the receptors *Frazzled* and *Uncoordinated-5* from 24 h after pupal formation onwards. We observe that overexpression of *NetB* causes severe β lobe fusion in the MB, which is similar to the MB defects seen in the *Drosophila* model of fragile X syndrome (FXS). Our results further show that fragile-X mental retardation protein FMRP inhibits the translational activity of human ortholog *Netrin-1* (*NTN1*). Knock-down of *NetB* significantly rescues the MB defects and ameliorates deficits in the learning and memory in FXS model *Drosophila*. These results indicate a critical role for *NetB* in MB lobe extension and identify *NetB* as a novel target of FMRP which contributes to learning and memory.

**Electronic supplementary material:**

The online version of this article (10.1186/s13041-019-0472-1) contains supplementary material, which is available to authorized users.

## Introduction

To form a complete and functional nervous system, neurons need to extend axons to reach specific targets. Proper axon guidance and extension are controlled by neuronal cell surface receptors and their extracellular ligands, known as axon guidance molecules [1–3]. The mushroom body (MB) neuropils in *Drosophila melanogaster* are a powerful model system for investigating axonal guidance and extension because of the unique structure of the MB [4–7]. During development, MB neurons, called Kenyon cells, experience a sequential differentiation process into three neuronal sub-types: α/β neurons, α′/β′ neurons and γ neurons. The cell bodies of these MB neurons form a pair of quadruple clusters in the dorsal posterior cortex and project their axons through an axon tract called the peduncle to the anterior region. The axons bifurcate into two branches at the anterior end of the peduncle and segregate into medial (β, β′ and γ) and dorsal (α, α′) lobes. Many axonal guidance cues are known to regulate development of the MB lobes or even sister branch-specific development. For example, Robo2/3 signaling mainly regulates dorsal and medial lobe extension, while Sema-1a signaling directs lobe outgrowth and orientation in a lobe- and axon branch-specific manner. Likewise, Eph signaling guides specific axon branches of MB neurons [3, 4, 6, 8]. As essential chemotropic cues for axon guidance during neural development, netrins are also expressed in the MBs [3], but their function in MB development is not yet clear.

Netrins are a family of laminin-related proteins that function as chemotropic guidance cues for migrating cells and axons during neural development [9, 10]. In *Drosophila*, two *Netrin* homologs have been identified, known as *NetA* and *NetB*. As key axonal guidance molecules, detailed studies have been conducted to investigate their roles in ventral nerve cord (VNC) development. Both *NetA* and *NetB* are highly expressed by the cells of the VNC midline, mainly guiding commissural axons either toward or away from the midline. In *Drosophila*, Frazzled (Fra), Uncoordinated-5 (Unc-5) and Down syndrome cell adhesion molecule (Dscam) have been identified as the three main receptors of netrins. During VNC development, Fra is involved in the attraction of commissural axon towards the midline, while Unc-5 acts to prevent commissural axons from crossing the midline. Dscam serves as an attractive receptor which acts in parallel with Fra during VNC development, but it can also respond to multiple other as-yet-unidentified ligands [11–14]. During MB development, Dscam is required for MB axon sister branch segregation [15–17], but it is less clear whether Fra and Unc-5 regulate MB development.

As a prominent structure for higher-order functions in the *Drosophila* protocerebrum, the MB is essential for learning and memory, similar to the hippocampus in mammals [3, 18]. *Drosophila* with MB defects exhibit impaired memory formation and deficits in sleep homeostasis. Fly models of cognitive disorders, such as fragile X syndrome (FXS) or Alzheimer’s disease, or of inherited cognitive deficits, for example caused by mutation of *ZC3H14*, show subtle MB defects [18–27]. FXS, the most common form of inherited monogenic disorder caused by mutation of the *fragile-X mental retardation 1* (*Fmr1*) gene, leads to transcriptional silencing of its encoded fragile-X mental retardation protein FMRP [28–30]. In *dfmr1* mutant flies, the MB is characterized by the failure of the β lobes to stop at the brain midline. Research by both McBride et al. and Chang et al. has demonstrated that MB defects can be rescued and courtship activity and associated memory can be restored by treatment with metabotropic glutamate receptor (mGluR) antagonists or GABAergic inhibitors [23–25]. These findings highlight the importance of structural integrity of the MB to learning and memory.

In our study, we focused on the roles of netrins in *Drosophila* MB development. By loss- and gain-of-function experiments we demonstrate that MB axons display lobe-specific NetB signaling, regulating lobe axon outgrowth. Fra and Unc5 were found to participate in lobe extension via genetic interaction with NetB signaling. Overexpression of *NetB* results in severe β lobe fusion similar to the MB abnormality observed in *dfmr1* mutant models, with increased *NetB* protein levels observed in the brains of *dfmr1* mutants. We further report that *NetB* human ortholog *NTN1* mRNA physically interacts with FMRP, and that *NTN1* mRNAs exhibit abnormal polyribosome profiles. Importantly, MB and memory defects are ameliorated in FXS *Drosophila* by knock-down of *NetB*.

## Materials and methods

### Drosophila stocks

Flies were cultured on standard *Drosophila* yeast-cornmeal molasses food at 25 °C. The following RNAi lines were obtained from the Vienna *Drosophila* Resource Center for use as stocks: UAS-*NetB*-RNAi (VDRC330183), UAS-*NetA*-RNAi (VDRC108577), and UAS-*Dscam1*-RNAi (VDRC108835). The following lines were obtained from the Bloomington *Drosophila* Stock Center: *NetA*^*Δ*^ (BDSC66878), *NetB*^*Δ*^ (BDSC66879), *NetB*^*tm*^ (BDSC66880), *Dscam1*^*1*^ (BDSC5934), *Fra*^*3*^ (BDSC8813), *Unc-5*^*MI04273*^ (BDSC37426), UAS-*Fra*-RNAi (BDSC40826), UAS- *Unc-5*-RNAi (BDSC33756), UAS-*mCD8-GFP* (BDSC5130), *dfmr1*^*Δ3*^, *dfmr1*^*50M*^ along with *Elav*-Gal4, *OK107*-Gal4, *repo-*Gal4 and other balancer flies. UAS-*NetA* and UAS-*NetB* lines were constructed and the expression level of *NetA* and *NetB* were verified.

### Antibodies and immunodetection

For indirect immunofluorescent staining, larval and adults brains were dissected, fixed and stained as previous described [31]. The following antibody dilutions were used: monoclonal 1D4 antibody (anti-FasII), 1:20 (Developmental Studies Hybridoma Bank, Iowa city, IA, USA); c-myc (9E10, anti-c-myc), 1:20 (Santa Cruz, TX, USA); Cy3-labeled anti-mouse, 1:200 (Jackson Immunoresearch, PA, USA). Confocal imaging was performed using a confocal microscope (TCS SP5; Leica Microsystems, Wetzlar, Germany). Defects in lobe length and width were defined by visually inspecting all brains. Only obvious and unambiguous differences in length or width were considered [3, 5].

For western blotting (WB), the protein quantity of each sample was estimated by the Bradford assay (Thermo Fisher Scientific, MA, USA), before being subjected to SDS-PAGE. The primary antibodies were diluted as follows: c-myc (9E10), 1:50 (Santa Cruz, TX, USA); β-actin, 1 μg/ml (Abcam, UK), anti-FMRP (1C3): 1:500 (Millipore, MA, USA).

### RNA immunoprecipitation (RNA-IP)

Immunoprecipitation was performed according to the modified protocol of Gross et al. [32]. Lysates from HEK293 cells for RIP were prepared in lysis buffer (20 mM Tris-HCl, pH 7.5; 150 mM NaCl; 5 mM MgCl_2_; 1 mM DTT; and 1% Triton X-100, supplemented with proteinase and RNase inhibitors) on ice. The solution was then precleared with 180 μl Dynabeads protein G (Thermo Fisher Scientific, MA, USA) for 2.5 h. Next, one third of the supernatant was saved as the input fraction for WB. The rest of the supernatant was then incubated with antibody-bound Dynabeads Protein G, blocked beforehand with anti-FMRP antibody (1C3) or normal mouse IgGs, overnight at 4 °C. Then, one sixth of the beads were boiled prior to WB. Total RNA from the remaining beads was extracted with an equal amount of *C. elegans* RNA added into the reaction mix. The RNA extract underwent reverse transcription, followed by quantitative real-time PCR (RT-qPCR). Relative values were calculated using the ddCt method with *18S rRNA* from *C. elegans* as an external control gene.

### Liner sucrose gradient fraction

Cultured normal and *fmr1* mutant lymphoblastoid cells were incubated with cycloheximide (100 μg/ml) or 30 mM EDTA at 37 °C for 30 min to arrest polyribosome migration. Cells were lysed using lysis buffer (10 mM HEPES-KOH, pH 7.5; 150 mM KCl; 10 mM MgCl_2_; 1 mM DTT; 100 μg/mL cycloheximide; and 1% Triton X-100, supplemented with proteinase and RNase inhibitors). Cytoplasmic extracts were loaded on a 15–60% (wt/vol) sucrose gradient and centrifuged at 45,000 rpm in a Beckman SW-55 rotor (Beckman Coulter, CA, USA) for 60 min at 4 °C to separate them into 10 fractions. Each fraction was isolated and analyzed by RT-qPCR [33], with an equal amount of *C. elegans* RNA used as an external control.

### Behavioral training and testing

The behavioral training and testing were performed as described previously [24, 25]. Virgin male flies were collected within 3 h of eclosion. Each male fly was collected in an individual food tube. Virgin *W1118* females were collected and kept in food tubes in groups of 10. Flies were aged for 5 d in a 12/12 h light/dark cycle at 25 °C before behavioral training and testing. All testing was performed between 11 am and 3 pm during the relative light phase with comfortable humidity. Mated females were 5 d old and were observed to mate with a male the night before training. All male flies were transferred to fresh food tubes the day before testing. Male flies were assigned randomly to groups for behavioral training and testing in a polystyrene chamber (8 × 10 × 12 mm), with the behavioral training and testing performed blind to group [24, 34, 35]. The total amount of time that a male spent on courtship activity when paired with an unanesthetized female target was scored either during a test period of 10 min or until successful copulation. The courtship index (CI) was measured as the percentage of total time spent courting during the observation [34].

#### Pavlovian olfactory learning and memory

Flies were trained with the classical conditioning procedure from Tully and Quinn [36]. After one training cycle, the olfactory learning (3 min-memory) was tested. The experiments were carried out at 25 °C and 70% humidity. Flies were collected after eclosion and incubated for 5 days before testing learning.

### Statistical methods

To assess lobe length, samples were obtained, with experimenters blind to genotype. Each α/β lobe of a MB was categorized into “normal”, “short” and “overextended” classes. For “normal”, the MBs show a paired neuropil structure in which the α lobes project toward the dorsal surface, while the β and γ lobes project toward the midline of the brain. For “short”, the MBs exhibit obviously reduced or absent lobes. “Overextended α lobes” is defined as overextension of the tip of the dorsal lobes toward the interhemispheric region, “overextended β lobes” as the β lobes cross the MB midline and fused, and “overextended γ lobes” as γ lobes project dorsally outside the normal axis due to overextension [6, 8]. The phenotypic severity in each group was quantified as the percentage of MBs that belong to the defective class, statistical significance was assessed by Fisher’s exact test. ANOVAs were performed for comparison between multiple groups. The other data were analyzed using Student’s two-tailed *t*-test.

## Results

### *Drosophila NetB* regulates MB lobe extension

**Fig. 1:**
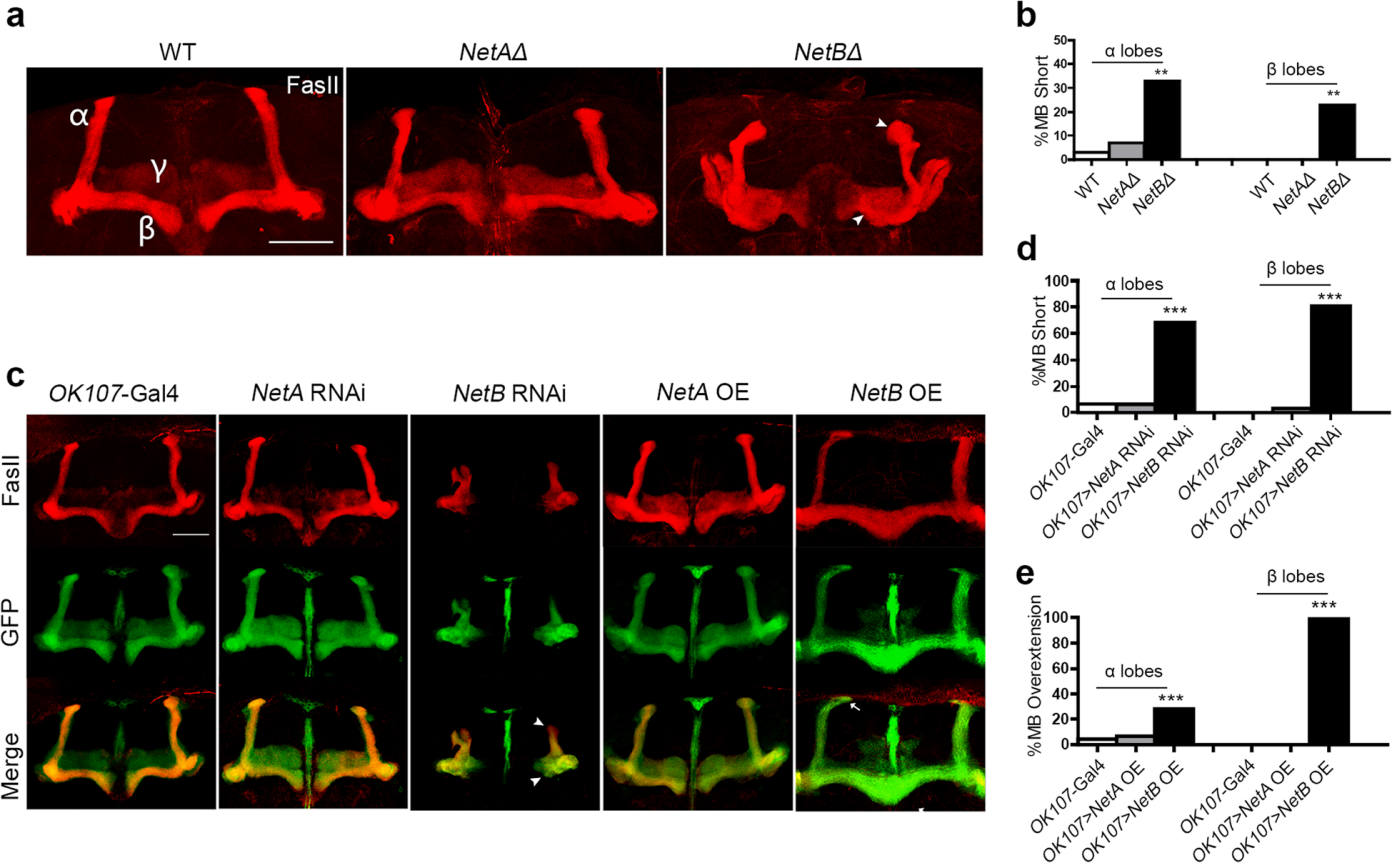
*NetB* regulates MB lobe extension. **a** Images of WT, *NetAΔ* and *NetBΔ* MBs in adult brains. FasII labeling showed similar lenghth of α/β lobes in WT and *NetA*^*Δ*^, whrease *NetB*^*Δ*^ MBs displayed short lobes (arrowhead). **b** Results from (**a**) were quantified for the percentage of brain hemispheres with short α/β lobes. (WT, *n* = 24; *NetAΔ*, *n* = 28; *netBΔ*, *n* = 33; n indicates fly numbers. **p < 0.01). **c** MBs from control, *NetA* RNAi, *NetB* RNAi, *NetA* over-expression (OE) and *NetB* OE were visulized by *mCD8-GFP* driven by *Ok107*-Gal4 and immunostaining for FasII. Altered NetA expression caused no neuroanatomical MB defects, wherease *NetB* knocked-down MBs showed servere short lobes (arrowhead) and *NetB* OE MBs resulted in overextended α/β lobes (arrow).**d** The percentage of brain hemispheres with short α/β lobes in *NetA/NetB* RNAi flies. (*OK107-*Gal4, *n* = 30; *OK107 >* UAS-*NetA* RNAi, *n* = 32; *OK107 >* UAS-*NetB* RNAi, *n* = 26; ***p < 0.001). **e** The percentage of brain hemispheres with overextension of α/β lobes in flies over-expressing Netrins. (*OK107-*Gal4, *n* = 30; *OK107 >* UAS-*NetA*, *n* = 30; *OK107 >* UAS-*NetB*, *n* = 28, ***p < 0.001). Significance was determined by Fisher’s exact test. Scale bars: 50 μm

To investigate the roles of *Drosophila* netrins in MB development, we assessed the overall structure of the adult MB (dissected at 3 d after eclosion) in wild type (WT), *NetA* deletion mutant *NetA*^*Δ*^, and *NetB* deletion mutant *NetB*^*Δ*^ flies. Anti-Fasciclin (FasII) antibody labels α/β lobes strongly and γ lobes weakly in wild type (WT) strain *W1118* adult brains (Fig. 1a). The WT MB showed a paired neuropil structure in which the α lobes project toward the dorsal surface, while the β and γ lobes project toward the midline of the brain. No obvious defects were observed in *NetA*^*Δ*^ flies compared with WT flies. However, the stereotyped morphology of MB lobes was disrupted, leading to a relatively smaller MB structure with short lobes in *NetB*^*Δ*^ flies (Fig. 1a). We statistically analyzed the MB defects, focusing on α/β lobes, which are easy to visualize. The α/β lobes were significantly shorter in *NetB*^*Δ*^ flies compared to those in WT flies, with the percentage of short α and β lobes being 33 and 23% in the two groups, respectively (Fig. 1b, *p* < 0.01). We also documented the effects of NetB on α’, β’ and γ lobe length (Additional file 1: Table S1).

To further analyze how netrins regulate MB morphogenesis, we investigated the effects of knock-down and overexpression of *NetA* and *NetB* using the pan-MB neuroblast driver *OK107*-Gal4. Reductions or increases of *NetA* activity resulted in no obvious neuroanatomical MB defects compared with control. Knock-down of *NetB* in MB neurons resulted in significantly greater numbers of short lobes, reminiscent of the phenotype observed in *NetB*^*Δ*^ mutants. Overexpression of *NetB* resulted in slight overextension of the tip of the α lobe toward the interhemispheric region and severe fusion in the β lobe (Fig. 1c, d). Similar phenotypes were also observed using the pan-neuronal *elav*-Gal4 driver (Additional file 2: Figure S1). These results suggest that *NetB* can regulate the precise extension of MB lobes.

### *NetB* is highly expressed in MB and controls MB lobe morphogenesis from 24 h after pupal formation

**Fig. 2:**
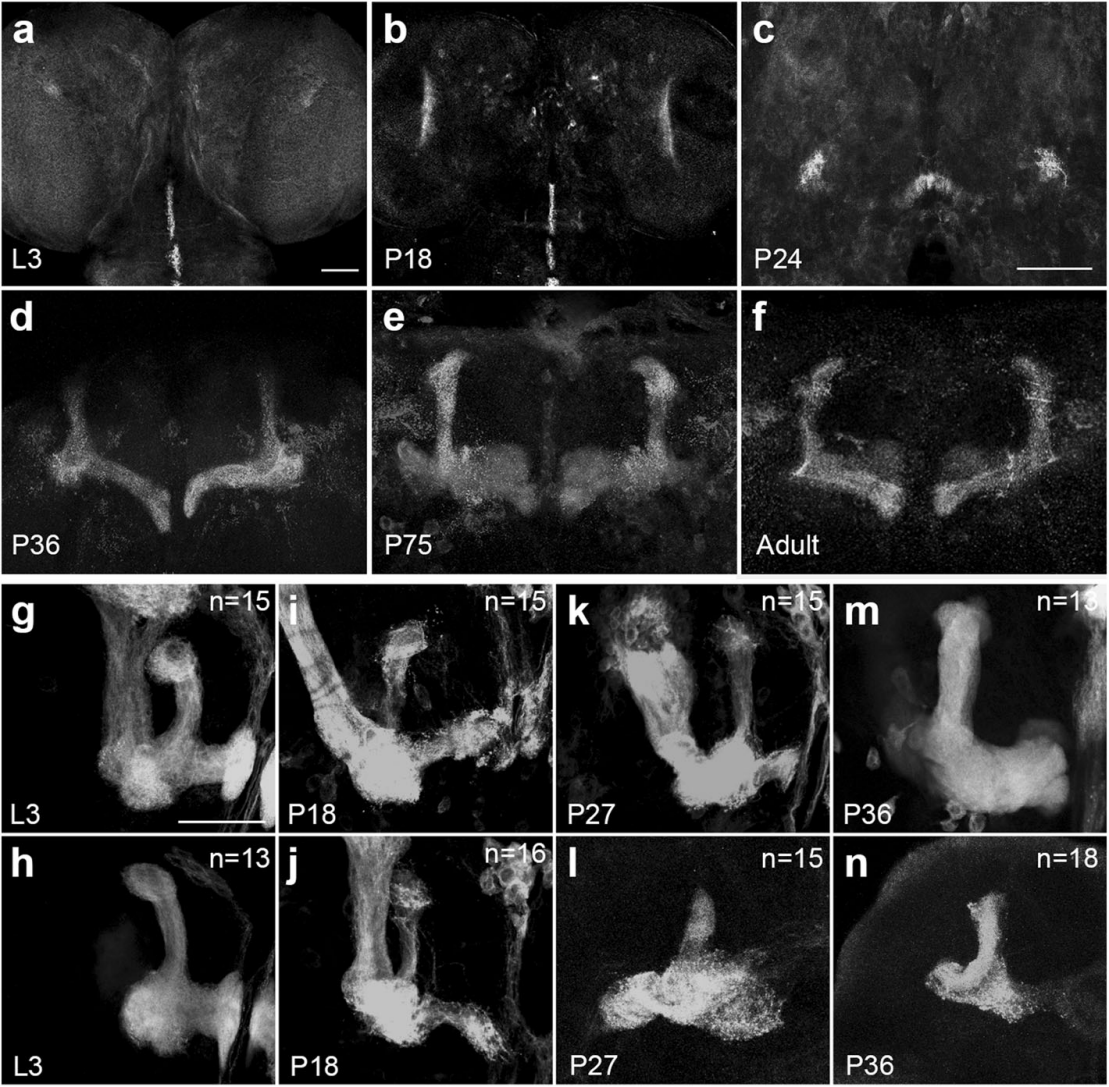
NetB expression pattern and MB phenotypes in *NetB* RNAi flies during brain development. **a**-**f** NetB expression pattern in *NetB*^tm^ flies, as revealed by anti-myc staining (gray). **a**-**b** Strong expression of NetB was observed in the VNC cells at third Larva stage (L3) and 18 h APF. **c** At stage of the 24 h APF, NetB was enriched around the end of peduncles of MB and in the ellipsoid body (EB). **d**-**f** NetB expression from 36 h APF until adult stage. MB lobes were strongly stained with anti-myc. **g-n** The MB morphology of the control (*OK107 >* UAS-*mCD8-GFP*) and *NetB* RNAi (UAS*-NetB* RNAi/+; UAS*-mCD8-GFP/+*;*OK107-*Gal4/+)flies at L3,18h APF,27 h APF,36 h APF. Upper line shows the control group MBs and lower line shows the *NetB* RNAi MBs. Numbers in the upper right corners indicate the numbers of observed MBs in corresponding genotypes

Next, we examined the expression pattern of *NetB* during *Drosophila* brain development. We labeled a myc-tagged membrane-tethered version of *NetB* (*NetB*^tm^), expressed under the control of the endogenous Netrin promoter [37, 38]. We observed that during the larval period and early pupal stage, *NetB* was mainly expressed in the ventral midline (Fig. 2a, b). From 24 h after pupal formation (APF), *NetB* was expressed in the ellipsoid body (EB) and around the end of peduncles (Fig. 2c). *NetB* was highly expressed in the MB lobes from 36 h APF into adulthood (Fig. 2d-f).

During MB development, both γ and α′/β′ neurons are generated and acquire similar projection patterns during the instar stage. After pupal formation, γ neurons undergo a dramatic reorganization and become restricted to the end of peduncle at around 24 h APF, while α′/β′ neurons remain relatively unchanged. Meanwhile, all MB neurons which are born after pupal formation become α/β neurons, and their axons extend to form the adult-specific lobes [39]. To determine the stage at which axonal defects start to be observed, we analyzed the morphology of MB lobes in *NetB* RNAi flies during metamorphosis. *NetB* RNAi lobes were indistinguishable from control at larval stage and at 18 h APF (Fig. 2g–j; *n* = 15 and *n* = 13 for control and *NetB* RNAi larval MBs, respectively; *n* = 15 and *n* = 16 for control and *NetB* RNAi 18 h APF MBs, respectively). Notably, at 27 h APF and 36 h APF, *NetB* RNAi MBs showed a much greater proportion of short lobes (Fig. 2k–n; *n* = 15 and 15 for control and *NetB* RNAi 27 h APF MBs, respectively; *n* = 13 and 18 for control and *NetB* RNAi 36 h APF MBs, respectively). These results imply that *NetB* is required for the MB lobe projection to mature after the completion of degeneration.

### Receptors Fra and Unc-5 are involved in MB development through genetic interaction with NetB

**Fig. 3:**
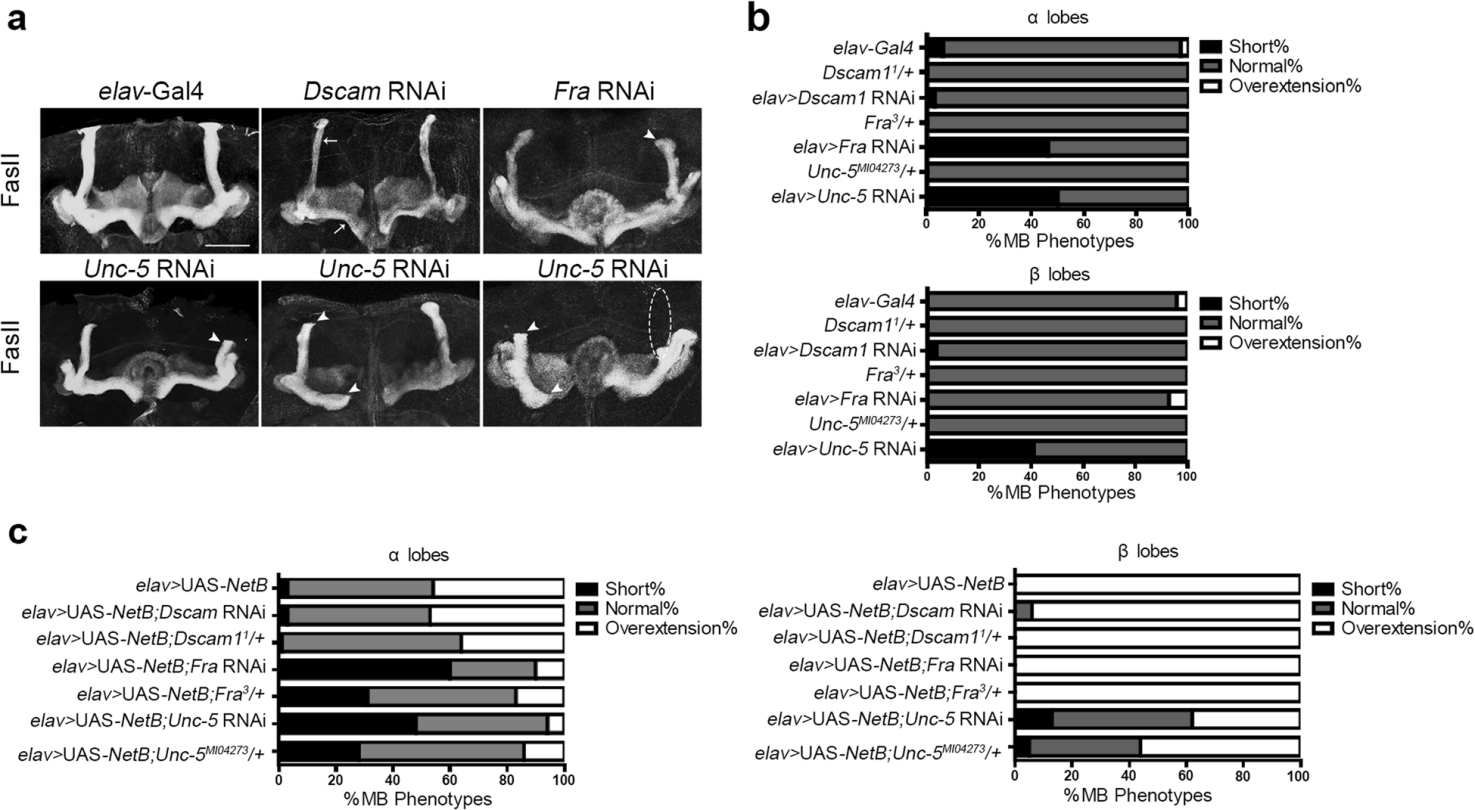
Receptors *Fra* and *Unc-5* show genetic interaction with *NetB* in MB lobe extension. **a** Receptors *Dscam*, *Fra* and *Unc-5* are involved in MB development. *Dscam* RNAi MBs displayed thin α/β lobes with normal length compared with control MBs; *Fra* RNAi MBs displayed short α lobes (arrowhead); Knock-down of *Unc-5* caused both short (arrowhead) or even missing (dashed lines) α/β lobes. **b** The percentage of brain hemispheres with short α/β lobes (*elav-*Gal4, *n* = 30; *elav > Dscam* RNAi, *n* = 30; *elav > Fra* RNAi, *n* = 28; *elav > Unc-5* RNAi, *n* = 34; ^***^p < 0.001; *Dscam1*^*1*^/+, *n* = 25; *Fra*^*3*^/+, *n* = 30; *Unc-5*^*MI04273*^/+, *n* = 30). **c** Receptors *Fra* and *Unc-5* show genetic interaction with *NetB*. The lobe phenotypes were classified as short, normal or overextension, and the quantification was shown as the percentages of brain hemispheres in each category. The percentage of overextended α lobes was significantly reduced in *elav* > UAS-*NetB*; *Unc-5* RNAi (or *Unc-5*^*MI04273/+*^) and in *elav >* UAS-*NetB*; *Fra* RNAi (or *Fra*^*3/+*^) compared with *NetB* OE (*elav* > UAS-*NetB*); The percentage of overextended β lobes was significantly reduced only in *elav* > UAS-*NetB*; *Unc-5 RNAi* (or *Unc-5*^*MI04273/+*^) lines. (*elav*-Gal4/Y; UAS-*NetB/+*, *n* = 30; *elav*-Gal4/Y; UAS-*NetB/+*; *Unc-5* RNAi/+, *n* = 48; *elav*-Gal4/Y; UAS-*NetB/ Unc-5*^*MI04273*^, *n* = 36; *elav*-Gal4/Y; UAS-*NetB/+*; *Fra*RNAi/+, *n* = 24; *elav*-Gal4/Y; UAS-*NetB/Fra*^*3*^, *n* = 36; *elav*-Gal4/Y; UAS-*NetB/+*; */Dscam* RNAi, *n* = 30; *elav*-Gal4/Y; UAS-*NetB/Dscam1*^*1*^, *n* = 34; *p* < 0.0001). Significance was determined by Fisher’s exact test. Scale bars: 50 μm

Dscam, Fra, and Unc-5 have been shown to function as netrin receptors during VNC development in *Drosophila* [11, 13, 40]. We investigated the possible roles of these receptors in MB formation. We made use of RNAi constructs targeting each receptor which were driven by elav-Gal4. Knock-down of *Dscam1* resulted in very thin and faint α/β lobes, but had no effect on lobe length (*n* = 30). Knock-down of *Fra* led to a significantly greater proportion of short α lobes, but no β lobe defects (*n* = 28). After knock-down of *Unc-5*, the dorsal or medial lobes were partially or completely lost compared with control (*n* = 34) (Fig. 3a, b). Furthermore, we also investigated the MB phenotypes of the receptor heterozygous mutant alleles (the mutants are often recessive lethal). Removing one copy of these receptors was not sufficient to cause any neuroanatomical defects in the MB on its own. Our data broadly indicate roles for *Dscam* in regulating divergent segregation of axons in the MB, *Fra* in α lobe outgrowth, and *Unc-5* in both α and β lobe extension.

We then examined whether NetB signaling affecting MB morphogenesis is dependent on these receptors. As overexpression of *NetB* results in overextended lobes, disruption of the NetB pathway may reduce these defects. We investigated the genetic interaction between netrin receptors and *NetB* by combining either a *Dscam1*^*1*^, *Fra*^*3*^ or *Unc-5*^*MI04273*^ allele (or an RNAi construct against one of these receptors) with elav-Gal4-driven *NetB* overexpression. Potential genetic interaction was quantified as the percentage of MB lobes categorized as short, normal, or overextended. We did not observe any significant alterations to the *NetB* overexpression phenotype when *NetB* overexpression was combined with mutations (or RNAi knock-down) in *Dscam*. Loss of one copy (or RNAi-mediated knock-down) of *Fra* reduced the percentage of α lobe overextension with no alteration to fused β lobe morphology. Introduction of *Unc-5*^*MI04273*^ (or RNAi-mediated knock-down of *Unc-5*) led to a robust decrease in both α and β lobe extension (Fig. 3c). Furthermore, we examined MB phenotypes in *NetB*^***Δ***^ homozygotes in combination with heterozygous mutations of receptor *Fra* and *Unc-5.* Introduction of these heterozygous alleles did not aggravate the phenotypes of *NetB* mutants, implying that *NetB* is the primary ligand responsible for regulating MB extension via Fra and Unc-5 (Table 1).

**Table 1:** No significant alteration of the *NetB*^*Δ*^ phenotypes in combination with mutations in *Fra* and *Unc-5* (Fisher’s exact test)

Genotypes	#brains	Lobe	% Short
NetB^Δ^/Y	32	α	33
		β	18
NetB^Δ^/Y; Fra^3^/+	25	α	32
		β	14
NetB^Δ^/Y; Unc-5^MI04273^/+	26	α	33
		β	15

### Genetic reduction of *NetB* rescues MB defects and ameliorates the learning and memory in *dfmr1* mutants

**Fig. 4:**
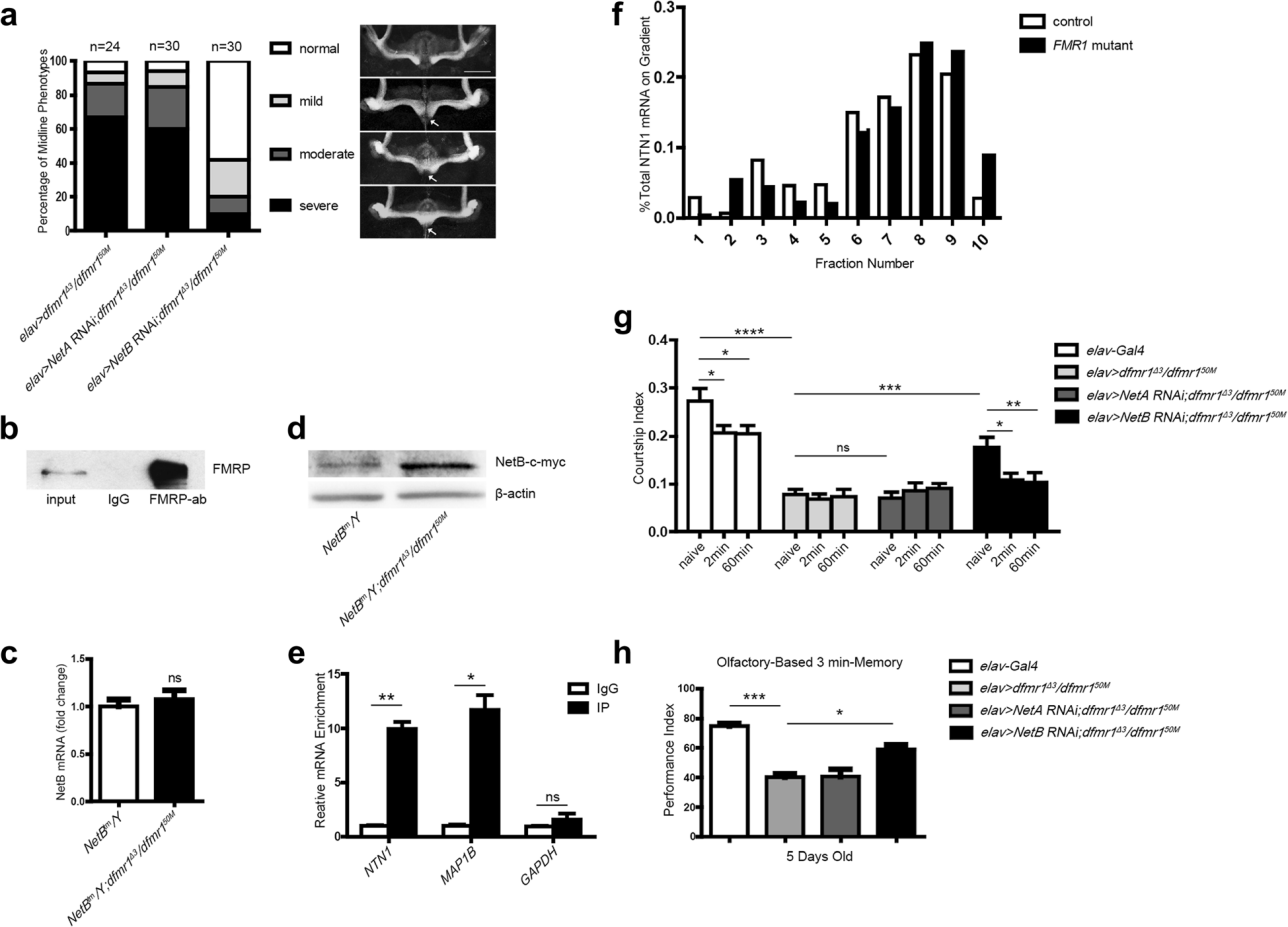
Knock-down of *NetB* rescues the MB defects and ameliorates the memory in *dfmr1* mutants. **a** Knock-down of *NetB* rescued the β lobe fusion in *dfmr1* mutants. **b**, **c** Western blot and RT-qPCR showed that NetB protein level was obviously elevated in the brains of *dfmr1* mutants while mRNA level was not significantly altered compared with control. **d**, **e** Interaction of FMRP with *NTN1* mRNA in HEK293 cells. **d** Western blot analysis showed FMRP was precipitated by the anti-FMRP antibody. IgG IPs were used as a negative controls. **e** RT-qPCR analysis indicated that *NTN1* and *MAP1B* (positive control) mRNAs were significantly enriched in anti-FMRP IPs. ^*^p < 0.05, ^**^p < 0.01. **f** Polyribosome profile of *NTN1* mRNA indicated a clear increase of the *NTN1* mRNA in the most actively translating ployribosomes in the *Fmr1* mutant lymphoblastoid cells compared with normal control (*N* = 3, **p < 0.01). **g** Knock-down of *NetB* in *dfmr1* mutants ameliorated the courtship activity, immediate recall memory and short-term memory of *dfmr1* mutant flies (^*^p = 0.0328, ^**^p = 0.0085, ^****^p < 0.0001, Two-way ANOVA). **h** Knock-down of *NetB* in *dfmr1* mutant flies by *elav-*Gal4 ameliorated the olfactory conditioning short-term memory of *dfmr1* mutants (^*^p < 0.05, ^***^p < 0.001, One-way ANOVA). Error bars = SEM

As a result of the deletion of dFMRP, *dfmr1* mutant flies exhibit deficits in cognitive function associated with a relatively high frequency of β lobes which cross the midline [24, 25, 41]. In our study, *NetB*-overexpressing flies exhibited similar defects as *dfmr1* mutants. We wondered whether MB defects are influenced by the NetB signaling pathway in *dfmr1* mutants. The severity and variability of β lobe midline crossing was classified in a *Drosophila* FXS model of *dfmr1*^*Δ3*^/*dfmr1*^*50M*^ trans-heterozygotes as either normal, mild, moderate or severe. The β lobes in *dfmr1* mutants cross over the midline and fuse at a fairly high frequency (93% total, with 67% severe, 19% moderate, 7% mild midline crossing; *n* = 24). The fusion percentage of β lobes after knock-down of *NetA* in *dfmr1* mutant flies did not differ significantly compared with *dfmr1* mutants (94% total, with 60% severe, 25% moderate, 9% mild midline crossing; *n* = 30). However, the fusion percentage was only 42% after knock-down of *NetB* in *dfmr1* mutants (with 10% severe, 10% moderate, 22% mild midline crossing; *n* = 30), indicating that reduction of *NetB* can significantly rescue MB defects in *dfmr1* mutants (Fig. 4a). Interestingly, a significant increase in NetB protein level was detected in the brains of *dfmr1* mutants with an unchanged total mRNA compared with control flies *NetB*^tm^ (Fig. 4b, c).

The canonical role of FMRP is translation suppression via direct mRNA-binding [42]. We performed RNA coimmunoprecipitation in cultured HEK293 cell extracts to investigate whether FMRP directly interacts with *NetB* human ortholog *NTN1* mRNA. As shown in Fig. 4d, FMRP was precipitated by the 1C3 anti-FMRP antibody from the immunoprecipitates (IPs), Next, we performed RT-qPCR on input, IgG and anti-FMRP Ab immunoprecipitated fractions to assess levels of NTN1 mRNA and control mRNAs. Notably, in comparison to the negative control IgG IPs group, *NTN1* mRNA was significantly enriched in the immunoprecipitated complexes. *MAP1B*, a known target of FMRP, was also significantly enriched in the anti-FMRP IPs. Glyceraldehyde-3-phosphate dehydrogenase (GAPDH) mRNA levels did not differ between groups, further demonstrating the specificity of the *NTN1*-mRNA-FMRP interaction (Fig. 4e). Next, a polyribosome profile was performed to test whether FMRP regulates *NTN1* mRNA translation. We used a lymphoblastoid cell line derived from an FXS patient who harbored a 237-kb deletion encompassing the entire *FMR1* and *FMR1NB* genes [42]. In normal lymphoblastoid cells, the samples treated by cyclohexamide resulted in the accumulation of stalled polysomes, and FMRP was distributed in every fraction. The samples treated by EDTA completely disrupted polysomes into ribosomal subunits, and FMRP was only distributed in messenger ribonucleoprotein and monosomal fractions (Additional file 3: Figure S2). We analyzed the polyribosome profile of *NTN1* mRNA in normal and *Fmr1* mutant lymphoblastoid cells treated by cyclohexamide. As shown in Fig. 4f, the majority of *NTN1* mRNA was associated with translating polyribosomes (fraction 6–10) in both normal control and *Fmr1* mutant cell extracts. Notably, there was a clear increase of *NTN1* mRNA in the most actively translating polyribosomes of *Fmr1* mutant cells (fractions 8–10) compared with normal cells (**p < 0.01). And the *NTN1* mRNA level was not altered in the mRNP/monosomes (fractions 1–5, *p* > 0.05) but showed a decrease in the fraction 6 and 7 (*p < 0.05) in the *Fmr1* mutant cells (Two-way ANOVA). These results indicate that *NTN1* translation is negatively regulated by FMRP.

Furthermore, we tested courtship-associated learning and memory ability in *dfmr1* mutant flies when knock–down of *NetB*. Learning and memory in *Drosophila* can be investigated experimentally by taking advantage of conditioned courtship behavior, as described previously. In conditioned courtship, courting *Drosophila* males perform characteristic behaviors: orienting toward the female; wing extension and vibration; licking; and attempting to copulate. Virgin females generally respond by mating, but recently mated females are unreceptive and will reject to copulate. A naïve male paired with a mated female will initially court her, but his courtship activity soon decreases. After 1 h of training with the mated female, his courtship activity remains depressed for 2 to 3 h when subsequently paired with a virgin female [24, 25]. As the results showed in Fig. 4g, *dfmr1* mutants showed low courtship interest in virgin females (naïve), impaired immediate recall memory at 0–2 min post-training, and a short-term memory deficit for up to 1 h, as previously reported [24, 25, 43]. A similar phenotype was also found during knock-down of *NetA* in *dfmr1* mutants. However, knock-down of *NetB* significantly ameliorated deficits in courtship activity, immediate recall memory and short-term memory.

Considering the olfactory conditioning short-term memory was impaired in *dfmr1* mutants [44], we asked whether knock-down of *NetB* in *dfmr1* mutants can also ameliorate the olfactory short-term memory. *dfmr1* mutants displayed significant defects in olfactory based 3 min-memory compared with normal control group (*elav-*Gal4), so did knock-down of *NetA* in *dfmr1* mutants. We found that knock-down of *NetB* can partially rescue the short-term memory of *dfmr1* mutant flies (Fig. 4h). The tests of locomotor and olfactory abilities in *dfmr1* mutant flies were replicated, showing no defects as previously reported [44, 45]. We also noticed that knock-down of *NetA* or *NetB* in *dfmr1* mutants showed no detectable changes in the locomotor and olfactory abilities (Additional file 4: Figure S3). Overall, we conclude that *NetB* regulates the learning and memory in the FXS *Drosophila* model.

## Discussion

This study has revealed a novel function for the conserved axonal guidance cue NetB which is responsible for regulating MB axon morphology during the development of higher-order brain centers in *Drosophila*. Although two *Drosophila* homologs of *Netrin* have been identified and share high homology, we demonstrate that *NetB*, but not *NetA*, regulates axon lobe extension of MB neurons. The amino acid sequence difference in the first EGF repeat of domain V might alter the receptor binding specificities of NetA and NetB [37, 46, 47], yet functional differences between NetA and NetB are still under debate. During embryonic nervous system development, NetA and NetB play similar roles in guiding commissural axon formation. When growing segmental nerve a (SNa) motor axons are repelled outwards from the central nervous system, only NetB is required to respond to the Unc-5 receptor. While ectopic expression of *NetA* appears to have no effect [37, 48]. These findings imply that they may play distinct roles in mediating axon guidance. The MB is a major source of NetB in the developing protocerebrum, with the precise regulation of MB lobe extension depending on the accurate distribution of *NetB* expression in space and time. In the current study, *NetB* exhibited a specific pattern of expression in the MB structure just after 24 h APF, when γ neuron degeneration was complete and axons had started to extend to form the mature lobes. Knock-down of *NetB* significantly impeded axon extensions at 27 h APF. These observations indicate that *NetB* is largely responsible for axon projection after degeneration which forms the adult-specific axon lobes.

In our study, we found that the Fra and Unc-5 receptors were involved in NetB signaling which regulates MB lobe extension, while Dscam showed no obvious genetic interaction with NetB. Dscam was involved in the mutual repulsion of sister branches, with the segregation accuracy of the sister branches depending on the repulsive force resulting from expression of the same set of Dscam isoforms. Loss of *Dscam* results in the failure of sister branch segregation, with flies exhibiting thin MB lobes [49]. *Fra* and *Unc-5* affect lobe length in distinct ways, as *Fra* is preferential for α lobes, whereas *Unc-5* affects the length of both α and β lobes. We wonder whether NetB from the MB cells interacts with the Frazzled and Unc5 of the same cells. *NetB* overexpression in the MB cells showed severe α and β lobe overextension, however, knock-down of *Unc-5* and *Fra* displayed no obvious MB defects and failed to ameliorate the defects of overextended α or β lobes when overexpressing *NetB* (Additional file 5: Figure S4a). Knock-down of *Fra* in glia didn’t alter MB morphology, whereas knock-down of *Unc-5* showed significant short MB lobes (Additional file 5: Figure S4b, c and Additional file 6: Table S2). These data imply that NetB from the MB cells interacts with the Unc-5 from neurons and glia surrounding the lobe fibers, and with the Fra from neurons surrounding the lobe fibers. This can be interpreted as possibly indicating a complex expression distribution of these receptors during MB development. The slit/robo2 and slit/robo3 signaling pathways provide a well-understood example of the complexity of regulation of MB lobe extension based on an intricate distribution of receptors. As Robo2 and Robo3 proteins are expressed in many domains in the developing central complex and in fibers which cross the central neuropil, they show elaborate and distinct expression distributions in these domains across space and time. Loss-of-function mutations to each specific receptor results in different MB lobe defects [8]. Thus, further research is needed to fully understand the different processes contributing to lobe formation which depend on the NetB/Fra or NetB/Unc-5 signaling pathways.

Overexpression of *NetB* in the MB results in severe β lobe fusion, resembling the defect seen in *dfmr1* mutants. As a translation suppressor binding to specific mRNAs, lack of FMRP results in dysregulated translation of many mRNAs, including those critical to neuronal development, plasticity, and dendritic spine architecture [30]. In this study, we identified *NTN1* mRNA as a specific target of FMRP. NetB protein levels were significantly enhanced in the brains of *dfmr1* mutants. Knock-down of *NetB* significantly rescued *dfmr1*-related MB defects and ameliorated deficits in short-term learning and memory in FXS model *Drosophila*. As a conserved axon guidance cue, NTN1 directs synaptic formation and synaptic plasticity during development [50]. Restricted sources of NTN1 can regulate the rapid local recruitment of synaptic proteins during neural development and spatially mediate the local translation of the synaptic proteome in *Aplysia* neurons for synaptic consolidation [50–53]. In the mature mammalian brain, NTN1 and its receptor DCC (an ortholog of Fra) are enriched at synapses, disruption of NTN1/DCC signaling reduces dendritic spine volume, severely attenuates LTP at hippocampal Schaffer collateral synapses, and impairs hippocampal-dependent learning and memory [54]. These findings suggest that overexpression of *NTN1* leads to synaptic structural defects, which may contribute to cognitive impairment in FXS.

In summary, we provide evidence for a significant regulation of NetB signaling in the MB lobe extension. We describe a novel FMRP target and find that knock-down of *NetB* can partially restore learning and memory in *dfmr1* mutants.

## Additional files

Additional file 1: **Table S1.** NetB affects α’/β’and γ lobe length (Fisher exact test). (DOCX 15 kb)

Additional file 2: **Figure S1. a-c**. Knock-down of *Netrins* with pan-neuronal driver *elav*-Gal4 showed similar phenotypes as driven by *OK107*-Gal4. **a**. MBs from control (*elav-*Gal4 > *mCD8*-*GFP*), *NetA* RNAi, *NetB* RNAi, *NetA* OE and *NetB* OE were visulized by *mCD8-GFP* driven by *elav*-Gal4 and immunostaining for FasII. The Control, *NetA* RNAi, *NetA* OE MBs showed normal structures, but knock-down of *NetB* showed short lobes (arrowhead). *NetB* OE MBs showed overextended lobes (arrow). **b**. The percentage of brain hemispheres with short α/β lobes in *NetA/NetB* RNAi flies. (*elav*-Gal4, *n* = 35; *elav > NetA* RNAi, *n* = 40; *elav* > *NetB* RNAi, *n* = 30; ***p < 0.001). **c**. The percentage of brain hemispheres with overextension of α/β lobes in flies over-expressing Netrins. (*elav*-Gal4, *n* = 35; *elav* > UAS-*NetA*, *n* = 30; *elav* > UAS-*NetB*, *n* = 30, ***p < 0.001). Significance was determined by Fisher exact test. Scale bars: 50 μm. (TIF 6225 kb)

Additional file 3: **Figure S2.** The distribution of FMRP in the normal lymphoblastoid cell extracts treated with cycloheximide and EDTA. After cycloheximide treatment, FMRP was distributed across all the fractions. However, in the EDTA treated samples, most of the FMRP was in fractions 1–5, which correspond to the free messenger ribonucleoprotein (mRNP) and monosomal fraction. (TIF 685 kb)

Additional file 4: **Figure S3.** Analysis of locomotor and olfactory abilities. **a**. Locomotor activity was measured by a line crossing assay [34]. All genotypes had similar locomotor activity profiles (*elav-*Gal4/Y, *elav-*Gal4/Y;*dfmr1*^*Δ3*^/*dfmr1*^*50M*^, *elav-*Gal4/Y; *UAS-NetA* RNAi/+;*dfmr1*^*Δ3*^/*dfmr1*^*50M*^, *elav-*Gal4/Y; *UAS-NetB* RNAi/+; *dfmr1*^*Δ3*^/*dfmr1*^*50M*^, for each genotype, we tested 20 flies). **b**. Olfactory capabilities were measured by the olfactory trap assay [35]. No differences were found between any of the genotypes tested with this assay at the 60 h time point. (TIF 6938 kb)

Additional file 5: **Figure S4.** *NetB* from the MB cells doesn’t interact with the Fra and Unc5 of the same cells. **a**. Knock-down of *Fra* and *Unc-5* with *NetB* overexpression in the MB cells failed to ameliorate the defects of overextended α and β lobes (*OK107* > UAS-*NetB*, *n* = 28; UAS*-NetB/+*; UAS-*Fra* RNAi/+; *OK107-*Gal4*/+*, *n* = 30; UAS-*NetB/+*; UAS-*Unc-5 RNAi/+*; *OK107*-Gal4*/+*, *n* = 28). **b**. Knock-down of *Fra* and *Unc-5* with *OK107-*Gal4 displayed normal α/β lobes with normal length. Knock-down of *Fra* by a glia specific driver *repo-*Gal4, the MBs showed normal structure. However, knock-down of *Unc-5* with *repo-*Gal4 caused short α/β lobes (arrowhead). **c**. The percentage of brain hemispheres with short α/β lobes (*OK107-*Gal4*, n* = 30; *repo-*Gal4, *n* = 28; *OK107 > Fra* RNAi, *n* = 25, *OK107 > Unc-5* RNAi, *n* = 20; *repo > Fra* RNAi, *n* = 20; *repo > Unc-5* RNAi, *n* = 22, ^***^p < 0.001). Significance was determined by Fisher’s exact test. Scale bars: 50 μm. (TIF 5078 kb)

Additional file 6: **Table S2.** The summary of the α/β lobe defects in knocking-down *Fra* or *Unc-5* by pan-neuron, MB and glia drivers respectively. (DOCX 15 kb)

## Abbreviations

APF: After pupal formation
CI: Courtship index
Dscam: Down syndrome cell adhesion molecule
EB: Ellipsoid body
Fmr1: Fragile-X mental retardation 1
FMRP: Fragile-X mental retardation protein
Fra: Frazzled
FXS: Fragile X syndrome
GAPDH: Glyceraldehyde-3-phosphate dehydrogenase
IPs: Immunoprecipitates
MAP1B: Micro-tubule associated protein
MB: Mushroom body
NetA: Netrin-A
NetB: Netrin-B
NTN1: Netrin-1
RNA- IP: RNA immunoprecipitation
SNa: Segmental nerve a
Unc-5: Uncoordinated-5
Unc-5: Uncoordinated-5
VNC: ventral nerve cord
