# Supplementary material for: Drosophila Netrin-B controls mushroom body axon extension and regulates courtship-associated learning and memory of a Drosophila fragile X syndrome model
Source: Mol Brain. 2019 May 28;12:52. doi: 10.1186/s13041-019-0472-1 (PMC6540430; doi:10.1186/s13041-019-0472-1)
Supplement: Supplementary file 1 — Table S1. NetB affects α’/β’and γ lobe length (Fisher exact test). (DOCX 15 kb) [file 13041_2019_472_MOESM1_ESM.docx]

**Additional file1: Table S1**. NetB affects α’/β’and γ lobe length ( Fisher exact test).

| Genotypes | # brains | Lobe | %short | %overextension |
| --- | --- | --- | --- | --- |
| *OK107-*Gal4 | 30 | α’ | 3 | 0 |
|  |  | β’ | 0 | 0 |
|  |  | γ | 0 | 0 |
| *OK107>NetA* RNAi | 32 | α’ | 3 | 3 |
|  |  | β’ | 0 | 0 |
|  |  | γ | 0 | 0 |
| *OK107>NetB* RNAi | 26 | α’ | 46*** | 0 |
|  |  | β’ | 73*** | 0 |
|  |  | γ | 58*** | 0 |
| *OK107>*UAS*-NetA* | 30 | α’ | 3 | 8 |
|  |  | β’ | 0 | 6 |
|  |  | γ | 0 | 0 |
| *OK107>*UAS*-NetB* | 28 | α’ | 0 | 64*** |
|  |  | β’ | 0 | 93*** |
|  |  | γ | 0 | 15* |

Knock-down of *NetB* in the MBs using *OK107-*Gal4 resulted in short α’/β’and γ lobes. Overexpression of *NetB* in the MBs by *OK107-*Gal4 resulted in overextended α’/β’and γ lobes. Knock-down or overexpression of *NetA* in MBs by *OK107-*Gal4 caused no significant defects of α’/β’ or γ lobes (Fisher’s exact test, **p*<0.05, ****p*<0.001).
