# Supplementary figures and images for: Drosophila Netrin-B controls mushroom body axon extension and regulates courtship-associated learning and memory of a Drosophila fragile X syndrome model
Source: Mol Brain. 2019 May 28;12:52. doi: 10.1186/s13041-019-0472-1 (PMC6540430; doi:10.1186/s13041-019-0472-1)

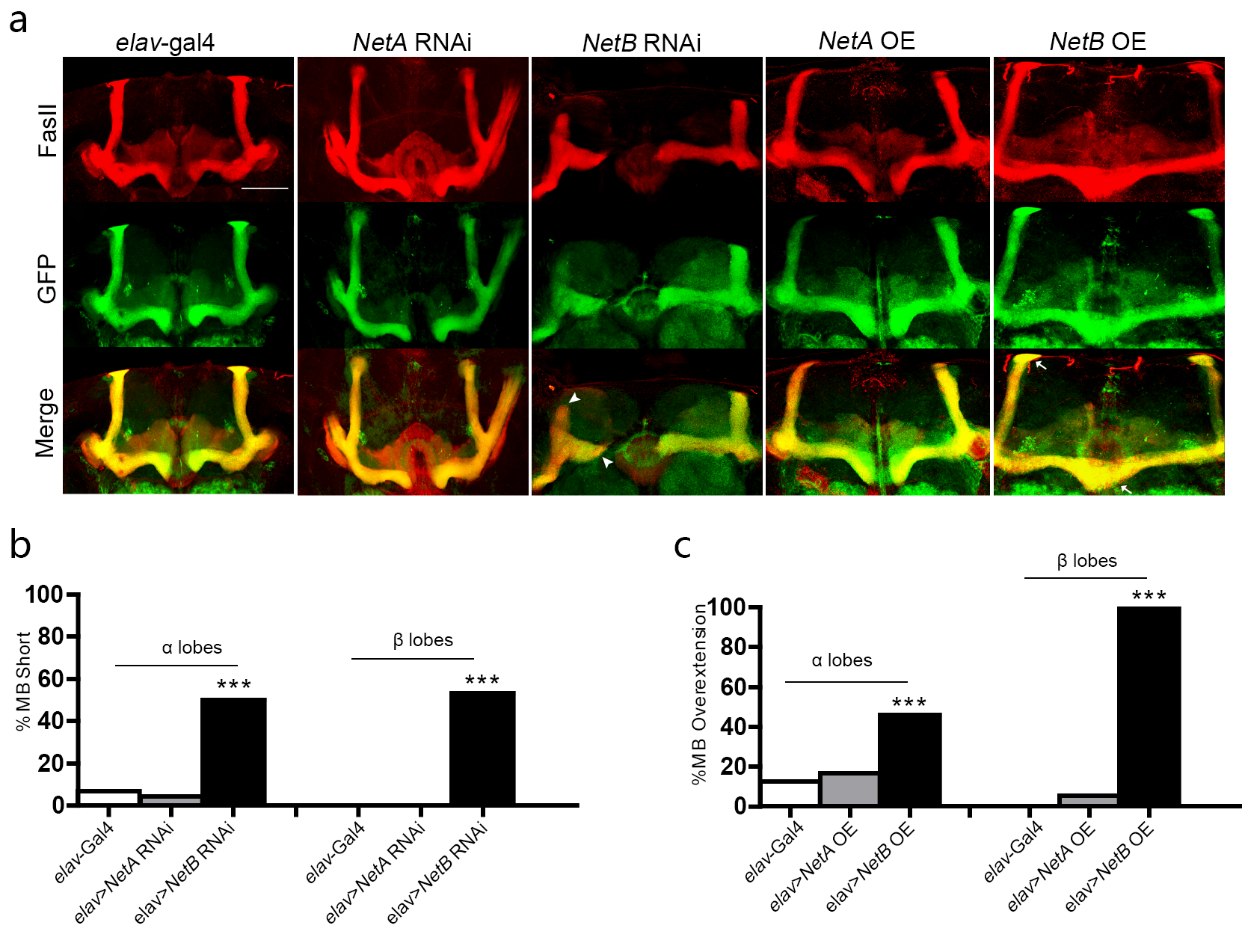

Supplement: Supplementary file 2 — Figure S1. a-c. Knock-down of Netrins with pan-neuronal driver elav-Gal4 showed similar phenotypes as driven by OK107-Gal4. a. MBs from control (elav-Gal4 > mCD8-GFP), NetA RNAi, NetB RNAi, NetA OE and NetB OE were visulized by mCD8-GFP driven by elav-Gal4 and immunostaining for FasII. The Control, NetA RNAi, NetA OE MBs showed normal structures, but knock-down of NetB showed short lobes (arrowhead). NetB OE MBs showed overextended lobes (arrow). b. The percentage of brain hemispheres with short α/β lobes in NetA/NetB RNAi flies. (elav-Gal4, n = 35; elav > NetA RNAi, n = 40; elav > NetB RNAi, n = 30; ***p < 0.001). c. The percentage of brain hemispheres with overextension of α/β lobes in flies over-expressing Netrins. (elav-Gal4, n = 35; elav > UAS-NetA, n = 30; elav > UAS-NetB, n = 30, ***p < 0.001). Significance was determined by Fisher exact test. Scale bars: 50 μm. (TIF 6225 kb) [file 13041_2019_472_MOESM2_ESM.tif]

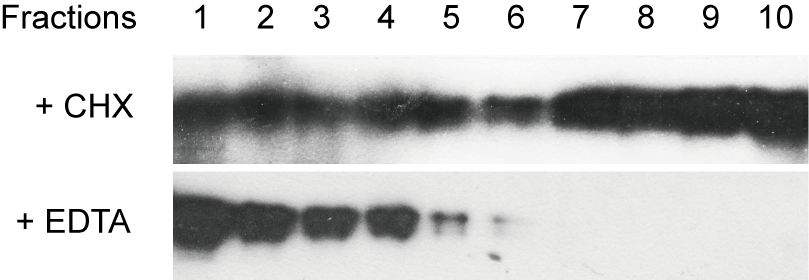

Supplement: Supplementary file 3 — Figure S2. The distribution of FMRP in the normal lymphoblastoid cell extracts treated with cycloheximide and EDTA. After cycloheximide treatment, FMRP was distributed across all the fractions. However, in the EDTA treated samples, most of the FMRP was in fractions 1–5, which correspond to the free messenger ribonucleoprotein (mRNP) and monosomal fraction. (TIF 685 kb) [file 13041_2019_472_MOESM3_ESM.tif]

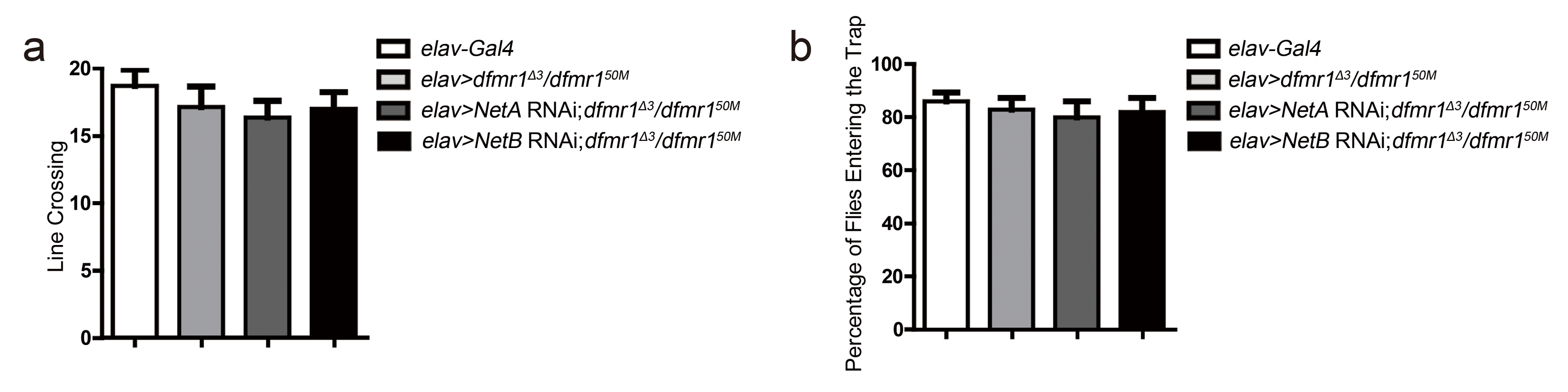

Supplement: Supplementary file 4 — Figure S3. Analysis of locomotor and olfactory abilities. a. Locomotor activity was measured by a line crossing assay [34]. All genotypes had similar locomotor activity profiles (elav-Gal4/Y, elav-Gal4/Y;dfmr1Δ3/dfmr150M, elav-Gal4/Y; UAS-NetA RNAi/+;dfmr1Δ3/dfmr150M, elav-Gal4/Y; UAS-NetB RNAi/+; dfmr1Δ3/dfmr150M, for each genotype, we tested 20 flies). b. Olfactory capabilities were measured by the olfactory trap assay [35]. No differences were found between any of the genotypes tested with this assay at the 60 h time point. (TIF 6938 kb) [file 13041_2019_472_MOESM4_ESM.tif]

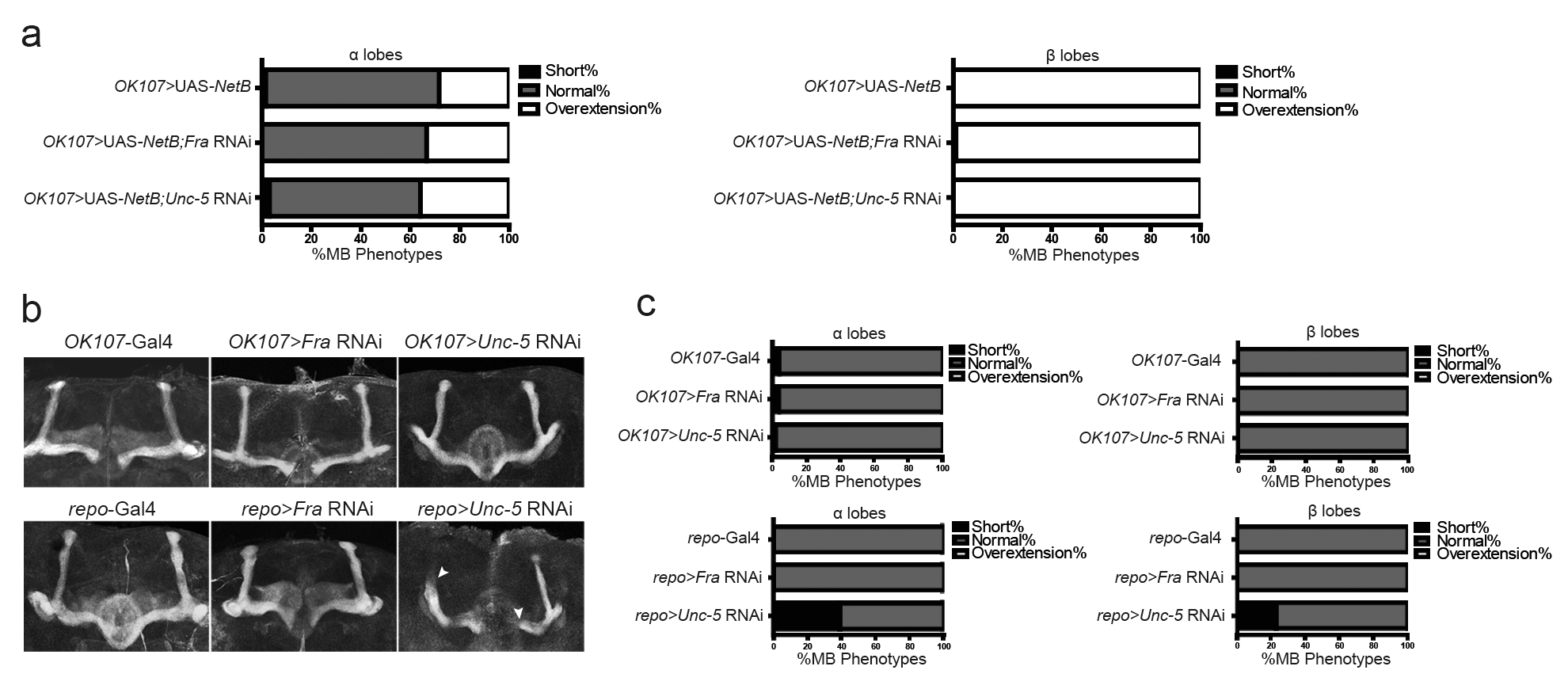

Supplement: Supplementary file 5 — Figure S4. NetB from the MB cells doesn’t interact with the Fra and Unc5 of the same cells. a. Knock-down of Fra and Unc-5 with NetB overexpression in the MB cells failed to ameliorate the defects of overextended α and β lobes (OK107 > UAS-NetB, n = 28; UAS-NetB/+; UAS-Fra RNAi/+; OK107-Gal4/+, n = 30; UAS-NetB/+; UAS-Unc-5 RNAi/+; OK107-Gal4/+, n = 28). b. Knock-down of Fra and Unc-5 with OK107-Gal4 displayed normal α/β lobes with normal length. Knock-down of Fra by a glia specific driver repo-Gal4, the MBs showed normal structure. However, knock-down of Unc-5 with repo-Gal4 caused short α/β lobes (arrowhead). c. The percentage of brain hemispheres with short α/β lobes (OK107-Gal4, n = 30; repo-Gal4, n = 28; OK107 > Fra RNAi, n = 25, OK107 > Unc-5 RNAi, n = 20; repo > Fra RNAi, n = 20; repo > Unc-5 RNAi, n = 22, ***p < 0.001). Significance was determined by Fisher’s exact test. Scale bars: 50 μm. (TIF 5078 kb) [file 13041_2019_472_MOESM5_ESM.tif]
