# Supplementary material for: Drosophila Netrin-B controls mushroom body axon extension and regulates courtship-associated learning and memory of a Drosophila fragile X syndrome model
Source: Mol Brain. 2019 May 28;12:52. doi: 10.1186/s13041-019-0472-1 (PMC6540430; doi:10.1186/s13041-019-0472-1)
Supplement: Supplementary file 6 — Table S2. The summary of the α/β lobe defects in knocking-down Fra or Unc-5 by pan-neuron, MB and glia drivers respectively. (DOCX 15 kb) [file 13041_2019_472_MOESM6_ESM.docx]

**Additional file 6: Table S2**. The summary of the α/β lobe defects in knocking-down *Fra* or *Unc-5* by pan-neuron, MB and glia drivers respectively.

| Genotypes | #brains | Lobe | %short |
| --- | --- | --- | --- |
| *elav*-Gal4 | 35 | α | 6 |
|  |  | β | 0 |
| *OK107*-Gal4 | 30 | α | 4 |
|  |  | β | 0 |
| *repo*-Gal4 | 28 | α | 4 |
|  |  | β | 0 |
| *elav*>*Fra* RNAi | 28 | α | 46 |
|  |  | β | 0 |
| *elav*>*Unc-5* RNAi | 34 | α | 50 |
|  |  | β | 41 |
| *OK107*>*Fra* RNAi | 25 | α | 4 |
|  |  | β | 0 |
| *OK107*>*Unc-5* RNAi | 20 | α | 2 |
|  |  | β | 0 |
| *repo*>*Fra* RNAi | 20 | α | 0 |
|  |  | β | 0 |
| *repo*>*Unc-5* RNAi | 22 | α | 29 |
|  |  | β | 23 |
